# Rapid Detection of Zeranol Contamination in Cereals Using a Quaternary Ammonium-Functionalized Terphen[3]arene-Based Optical Sensor

**DOI:** 10.3390/foods14050863

**Published:** 2025-03-03

**Authors:** Danni Wang, Mengyu Ye, Huijuan Yu, Kejing Niu, Chunju Li, Dong-Sheng Guo, Yuefei Wang

**Affiliations:** 1 State Key Laboratory of Chinese Medicine Modernization, Tianjin University of Traditional Chinese Medicine, Tianjin 301617, China; wangdn1220@163.com (D.W.); yemengyu2022@163.com (M.Y.); huijuanyu@tjutcm.edu.cn (H.Y.); 18317357226@163.com (K.N.); 2 Haihe Laboratory of Modern Chinese Medicine, Tianjin 301617, China; 3 Key Laboratory of Inorganic-Organic Hybrid Functional Material Chemistry, Ministry of Education, Tianjin Key Laboratory of Structure and Performance for Functional Molecules, College of Chemistry, Tianjin Normal University, Tianjin 300387, China; cjli@shu.edu.cn; 4 State Key Laboratory of Elemento-Organic Chemistry, Key Laboratory of Functional Polymer Materials (Ministry of Education), Frontiers Science Center for New Organic Matter, Collaborative Innovation Center of Chemical Science and Engineering, College of Chemistry, Nankai University, Tianjin 300071, China

**Keywords:** supramolecular sensing, zeranols, macrocycles, biphen[*n*]arenes, cereals

## Abstract

To ensure food safety and quality, sensitive and accurate methods for rapidly detecting mycotoxins have become imperative. Zeranols (ZERs) are a class of mycotoxins commonly found in cereals, posing serious health risks, including hormonal disruption and carcinogenic potential. In response to this pressing concern, we have developed a simple yet highly sensitive and high-throughput supramolecular sensing approach based on novel macrocycles known as extended biphen[*n*]arenes for monitoring ZERs in cereal matrices. The proposed approach utilizes the indicator displacement assay (IDA) and employs quaternary ammonium-functionalized terphen[3]arene (CTP3) as the host molecule for capturing ZERs. This method achieves a highly sensitive response, owing to the robust synergistic interactions between CTP3 and ZERs, ensuring the reliable detection of these harmful compounds. Significantly, not only does the established approach provide an alternative strategy for inspecting high-risk grains contaminated by ZERs, but it also demonstrates novel applications of biphen[*n*]arenes for mycotoxin detection.

## 1. Introduction

Crops are susceptible to contamination with mycotoxins during cultivation, processing, storage, and distribution, resulting in a large amount of wasted resources and serious financial loss. A total of 25% of crops worldwide are affected by mycotoxins annually, which leads to direct or indirect losses of billions of dollars [1]. Zeranols (ZERs) belong to a group of non-steroidal estrogenic mycotoxins produced by Fusarium, mainly including zearalanone (ZAN), α-zearalenol (*α*-ZOL), *α*-zearalanol (*α*-ZAL), *β*-zearalenol (*β*-ZOL), *β*-zearalanol (*β*-ZAL), and zearalenone (ZEN) [2]. ZERs are extremely harmful to human health because of their estrogenic activities, which cause genital lesions and reproductive disorders by competitively binding to estrogen receptors [3]. Beyond reproductive toxicity, ZERs exhibit hepatotoxicity, genotoxicity, immunotoxicity, and carcinogenicity, with the IARC classifying it as a group III carcinogen [4,5,6]. To monitor ZERs in complex matrices such as foods, feeds, and medicinal materials, a simple, rapid, and sensitive analytical method is urgently required for early warning and prevention of ZER production. Currently, widely used detection methods for ZERs include high-performance liquid chromatography equipped with a fluorescence detection system (HPLC–FLD) [7], liquid chromatography–tandem mass spectrometry (LC–MS/MS) [8], immunoassay [9], molecular imprinting [10], biosensors [11], etc. As the classic methods, LC–MS/MS and HPLC–FLD are employed in national and regional standards for their high sensitivity and reproducibility [12,13,14]. As technology advances, immunoassays, molecular imprinting, and biosensors gradually show great potential due to their high throughput and practicality. Notably, these methods suffer from their constraints, including costly analytical instrumentation, complex and protracted procedural steps, and stringent experimental conditions.

The supramolecular sensing approach, underpinned by the indicator displacement assay (IDA), stands out as a facile, environmentally friendly, and high-throughput strategy for the detection of a wide array of analytes [15]. This method possesses the subtle yet profound molecular recognition behaviors that occur between the analytes and macrocycles, translating these microscopic interactions into readily measurable physical or chemical signals. The IDA technique not only affords a straightforward and rapid means of detection but also boasts simplicity and efficiency, allowing for swift analysis without the need for complex sample preparation or instrumentation. Additionally, its versatility allows for the customization and adjustment of combinations of macrocycles and indicators, enabling tailored detection methods based on analyte structures. This adaptability, combined with the high sensitivity and selectivity of the method, has positioned IDA-based supramolecular sensing as a powerful tool, offering a promising solution for a broad spectrum of analytical challenges in various fields [16,17]. The classic macrocycles evolved from crown ethers, cyclodextrins (CDs), and calixarenes (CAs) to cucurbiturils (CBs), both of which function as supramolecular sensors of biomarkers for the diagnosis of diseases [18,19], environmental pollutants [20], etc. However, classic macrocycles, such as CDs, CAs, and CBs, are characterized by their relatively small cavity sizes, single backbone structures, and restricted capacity for functional group modification at their peripheral sites, which constrain their ability to modulate the physicochemical properties of the macrocyclic framework significantly. As an important task for supramolecular sensing, designing and synthesizing novel macrocycles with various backbones, sizes, geometries, and chemical environments is indispensable and urgently needed, which opens new possibilities for diverse analyte sensing [21,22]. Biphen[*n*]arenes (BAs) are a category of novel macrocycles composed of substituted biphenyl units linked by methylene bridges [23]. In view of the simple and modular synthetic characteristics, BAs with different topologies, adjustable sizes, and charge properties have been developed for the sensing of acetylcholine and choline [24], paraquat and diquat [25,26], alkaloids [27], organometallic salts, and other analytes, such as carboxylato-biphen[4]arene, carboxylato-biphen[3]arene, and 2,2′-biphen[4]arene, showing broad development space in the field of supramolecular sensing.

**Scheme 1:**
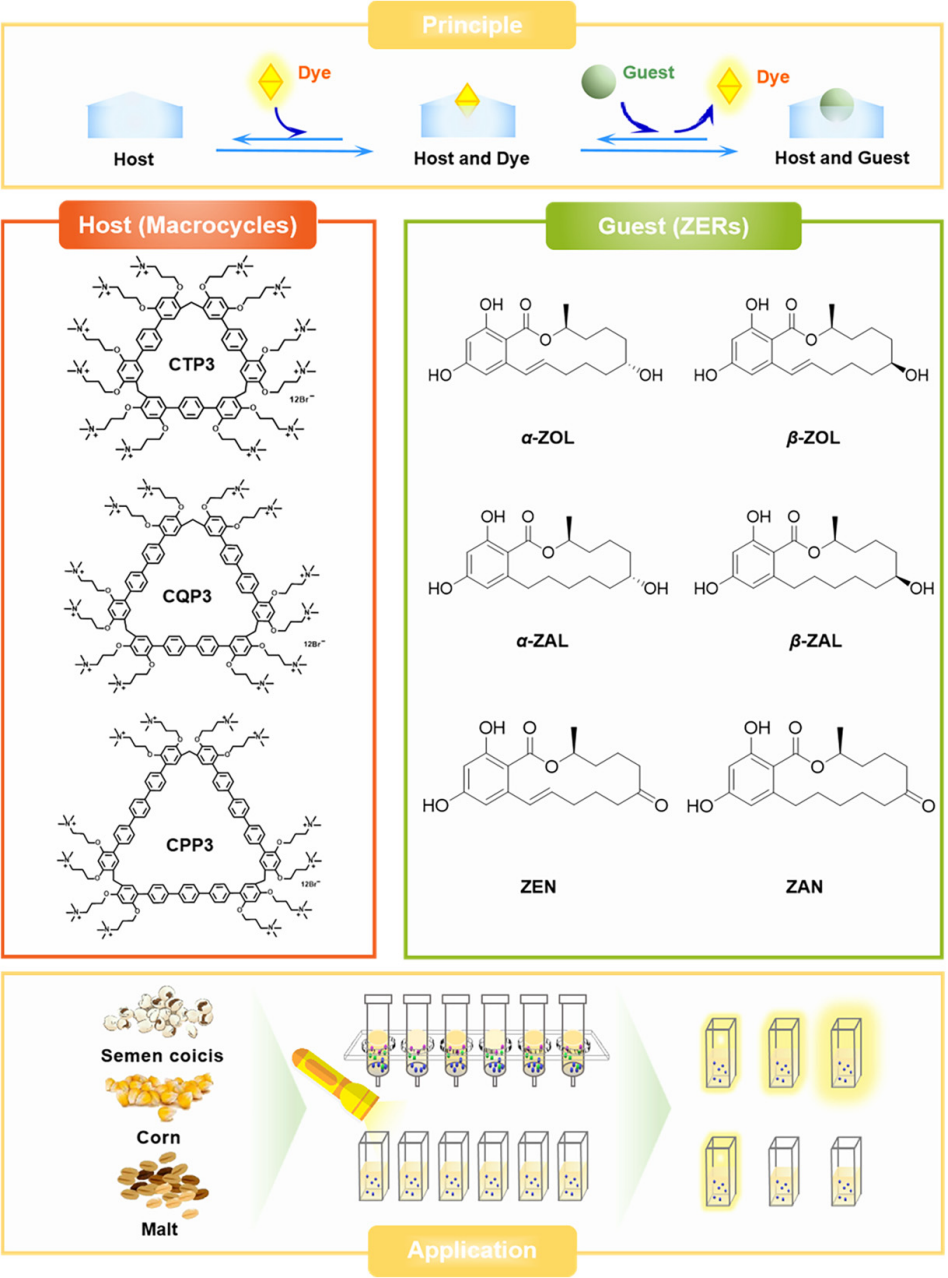
Schematic representation of monitoring ZER levels in cereals using supramolecular sensing.

Herein, based on quaternary ammonium-functionalized terphen[3]arene (CTP3), simple and sensitive supramolecular sensing for ZERs was investigated. Based on IDA, CTP3 showed a satisfactory association constant (*K_a_*) with ZAN, which is superior to other tested macrocycles (CDs, CBs, and CAs) (Scheme 1). Successively, the response consistency of CTP3 to ZERs (ZAN, *α*-ZOL, *α*-ZAL, *β*-ZOL, *β*-ZAL, and ZEN) was demonstrated using CTP3•fluorescein (CTP3•Fl) as the reporter pair. Subsequently, to gain deeper insight into the intricate molecular interaction dynamics, a comprehensive study of the molecular interaction patterns between CTP3 and ZERs was conducted by using density functional theory (DFT) calculations. In this study, the established supramolecular sensing method based on CTP3 was effectively applied for the rapid detection of ZERs in cereals (corn, Job’s tears, and malt), providing an alternate methodology for the timely warnings of cereals at high risk of contamination with ZERs and expanding the application scenario of biphen[*n*]arenes regarding the detection of mycotoxins and other toxic substances.

## 2. Materials and Methods

### 2.1. Reagents and Materials

ZEN was acquired from Hygiena Shanghai Trading Co., Ltd. (Shanghai, China). ZAN was manufactured by Witega Laboratory (Berlin, Germany). *α*-ZOL was purchased from the Panphy Chemicals Corporation (Rancho San Francisquito, La Quinta, CA, USA). *α*-ZAL was procured from Sigma-Aldrich Trading Co., Ltd. (Shanghai, China). *β*-ZAL and *β*-ZOL were supplied by the Laboratory of the Government Chemist (London, UK). From Shanghai Jizhi Biochemical Technology Co., Ltd. (Shanghai, China), formic acid was procured. Acetonitrile and methanol were secured from Tianjin Concord Technology Co., Ltd. (Tianjin, China). Fl and 8-hydroxypyren-1,3,6-trisulfonate (HPTS) were secured from Shanghai Yuanmu Biological Technology Co., Ltd. (Shanghai, China). HEPES and neutral red (NR) were purchased from Tianjin Heowns Biochem Technologies, LLC (Tianjin, China). The following substances were sourced from Sigma-Aldrich Co., Ltd. (St. Louis, MO, USA): Trans-4-[4-(Dimethylamino)styryl]-1-methylpyridinium iodide (DSMI), *β*-cyclodextrin (*β*-CD), *γ*-cyclodextrin (*γ*-CD), cucurbit[6]uril (CB[6]), cucurbit[7]uril (CB[7]), cucurbit[8]uril (CB[8]), rhodamine B (RhB), and acridine orange (AO). Methylene blue (MB) was procured from Merck KGaA Co., Ltd. (Darmstadt, Germany). As for *p*-sulfonatocalix[6]arene (SC6A), *p*-sulfonatocalix[5]arene (SC5A), *p*-sulfonatocalix[4]arene (SC4A), guanidinocalix[5]arene (GC5A), sulfonated azocalix[6]arene (SAC6A), and sulfonated azocalix[8]arene (SAC8A), they were synthesized as previously reported [28,29,30,31]. CTP3, quaternary ammonium-functionalized quaterphen[3]arene (CQP3), and pentaphen[3]arene (CPP3) were prepared according to our previously reported method [32]. AflaTest total immunoaffinity columns were supplied from Qingdao Senge Instrument Systems Co., Ltd. (Qingdao, China). Forty shipments of cereals, including 10 batches of corn (C1–C10), 10 batches of malt (M1–M10), and 20 batches of Job’s tears (JT1–JT20), were collected from markets in China.

### 2.2. Preparation of HEPES Buffer

By dispersing 2.33 g HEPES in 0.9 L purified water, a 10 mM HEPES buffer was prepared; then, we adjusted the pH to 7.4 with 0.1 M sodium hydroxide solution and added purified water to reach 1 L.

### 2.3. Preparation of the Sample Solutions for Testing

By consulting the National Food Safety Standard [12], 10 g sample powder, 2 g NaCl, and 50 mL of 90% acetonitrile were transferred into a 120 mL centrifuge tube to rapidly homogenize for 2 min and then centrifuged for 10 min. Then, in a 50 mL centrifuge tube, 10 mL supernatant was diluted with PBS buffer (40 mL) and centrifuged for 10 min to obtain the sample solution. An amount of 10 mL PBS buffer and 10 mL water were successively employed to wash the ZERs immunoaffinity column, and then 20 mL sample solution was passed through at 3 mL/min. After air-drying for 3 mL, analytes were eluted with 1.5 mL methanol at 3 mL/min for HPLC detection. Successively, the obtained elution (1 mL) was concentrated and subsequently reconstituted by 1 mL HEPES buffer, which was centrifuged in an ultrafiltration centrifuge tube (3 kD) at 8000 rpm for 5 min. The filtrate was transferred and diluted five times with HEPES buffer for supramolecular sensing.

### 2.4. HPLC Analysis

ZERs were quantified with a FLD detector-equipped HPLC e2695 (Waters, Milford, MA, USA). In methanol–acetonitrile–water (50:15:35, *v*/*v*) solution at 1.0 mL/min, the chromatographic separation was performed on a Waters XBridge C18 column (4.6 × 250 mm, 5 μM) at 30 °C. Sample solution (10 µL) was injected for analysis. FLD had excitation and emission wavelengths of 300 and 450 nm, respectively.

### 2.5. Fluorescence Spectroscopy

Stationary-state fluorescence spectra were recorded using the Varian Cary spectrometer (Agilent Technologies Inc., Santa Clara, CA, USA) equipped with a Varian Cary single-cell Peltier accessory in a 10 × 10 × 45 mm^3^ quartz cell (light path = 10 mm). In a 1:1 binding model, be it the host–guest binding stoichiometry in direct host–guest titrations or the competitive binding model in competitive titrations, the association constant (*K_a_*/M^−1^) was determined. The fitting modules were supplied by the “Fitting Functions” column of Prof. Nau’s group’s webpage (http://www.jacobs-university.de/ses/wnau) (accessed on 1 September 2018), which were introduced comprehensively in our published work [33]. Every experiment was run in HEPES buffer (10 mM, pH 7.4) at 25 °C.

### 2.6. Theoretical Simulations

Density functional theory calculations were all completed using Gaussian 16 [34]. For all computational analyses, a density-based solvation model (SMD) was employed [35]. The theoretical level adopted for geometry optimization is B3LYP/6-31G(d) in combination with Grimme’s D3 dispersion correction [36,37]. In Gaussian 16, the default convergence criteria were employed for geometry optimization. Multiwfn 3.8 software was used to perform an Independent Gradient Model based on Hirshfeld partition (IGMH) analysis and Molecular Electrostatic Potential (MEP) analysis [38]. Molecular graphs are shown using Visual Molecular Dynamics (VMD) software (version 1.9.3) [39].

### 2.7. Statistical Analysis

Three independent replicates were performed to give the mean ± standard deviation (s.d.). To show * *p* < 0.05, ** *p* < 0.01, and *** *p* < 0.001 as significance values, Mann–Whitney U tests were undertaken. By utilizing Spearman’s correlation coefficient, the correlation was assessed about various non-normally distributed variables. Bland–Altman analysis was chosen to assess the agreement of different measurements, and the mean bias and the 95% confidence interval (CI) of limits of agreement (LoAs) were quantified and computed.

## 3. Results and Discussion

### 3.1. Design and Application of Automated Enrichment Equipment for Mycotoxin

**Figure 1:**
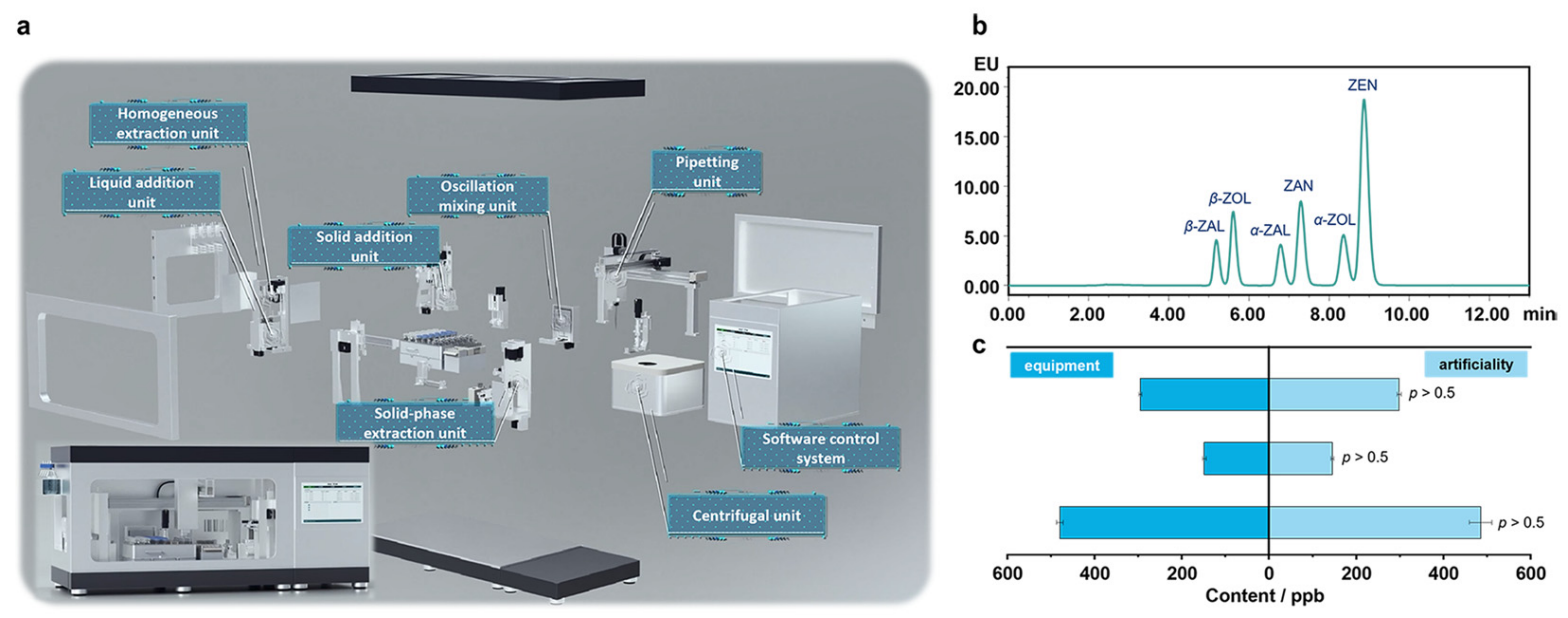
Design and application of automated enrichment equipment for mycotoxins. (**a**) The schematic diagram of automated enrichment equipment for mycotoxins. (**b**) HPLC chromatogram of ZERs. (**c**) The content of ZERs detected in three batches of Job’s tears by manual and automated methods.

Traditional mycotoxin enrichment methods include sample addition, liquid addition, extraction, centrifugation, transferring of sample solution, and enrichment, whose procedures are complicated, time-consuming, and prone to personal error, posing a great challenge to the realization of the high-throughput sample handling. Consequently, in order to conquer the obvious shortcomings, we have developed automated enrichment equipment for mycotoxin according to the procedures released by the Chinese Pharmacopoeia (ChP) for mycotoxin enrichment (Figure 1a) [13]. The equipment incorporates the latest sample preparation techniques and integrates various components to achieve automated handling for sample enrichment, such as a liquid addition unit, solid addition unit, homogeneous extraction unit, solid-phase extraction unit, pipetting unit, centrifugal unit, and operation software control system. The liquid addition unit provides online mixing of a solvent and oscillating mixing of a solvent and herb powder. The solid addition unit is responsible for the addition of particles (diameter, 0.01~4 mm), which is conducive to the extraction of interesting compounds. The homogenization unit is capable of homogenizing herb powder to greatly improve extraction efficiency. The pipetting unit is equipped with a 1 mL high-precision syringe pump, enabling precise pipetting. The software control system is designed to facilitate operations for laboratory users. The utilization of the equipment markedly enhances personnel efficiency, curtails solvent consumption, and guarantees precise and reproducible outcomes in comparison to manual labor.

To optimize the resolution and sensitivity for the detection of ZERs, the gradient elution conditions and detection wavelength for HPLC–FLD were fine-tuned based on the ChP method (Figure 1b). To verify the automatic pre-treatment ability of the developed equipment for mycotoxin enrichment, three batches of Job’s tears were processed using both manual and automated methods for ZER detection, which were demonstrated to have no significant difference, as illustrated in Figure 1c. Thus, the superiority of automated equipment has been conclusively demonstrated in minimizing the risk of mycotoxin exposure, eliminating manual labor, saving time, ensuring continuous operation, and providing reliability. This facilitates the achievement of high-throughput, straightforward, and reproducible sample processing.

### 3.2. Screening of Macrocycles Guided by Fluorescence Sensing for ZEN

**Figure 2:**
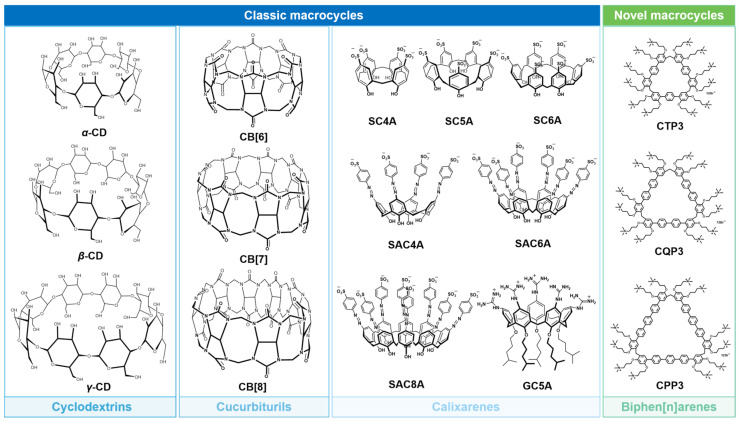
The chemical structures of classic and novel macrocycles.

According to an indicator displacement assay (IDA), the competitive analyte reversibly displaces the indicator from the host•indicator complex, resulting in a modulated optical response that translates the microscopic level of molecular recognition into a macro-level optical signal. To establish an optimal satisfactory association constant (*K*_a_) of analyte•host complexes based on IDA, classical and novel macrocycles were employed as the hosts to evaluate their binding ability with ZEN, including CDs, CAs, CBs, and BAs (Figure 2).

As environmentally friendly macrocycles, CDs are slightly pyramidal annular cavities formed by *α*-1,4-glycosidic units with a hydrophobic internal cavity and hydrophilic external surface [40,41]. CAs are cyclic oligomers consisting of several phenols linked by methylene in the vicinity of phenolic hydroxyl, allowing for more stable binding to a guest due to their tunable cavity structure, variable conformation, and multiple derivatization sites [42]. CBs are pumpkin-shaped cyclic macrocycles composed of multiple glycouril units bridged by methylene with upper and lower ends surrounded by negatively charged carbonyl oxygen [43,44]. Unfortunately, none of the classical macrocyclic hosts (CDs, CBs, CAs) can realize ZEN sensing (Table 1, Figures S1–S9). Overall, the CBs, CDs, and CAs cannot form highly affinity-selective host–guest complexes with ZEN, stemming from their unsuitable skeletal structure and cavity micro-environment.

**Figure 3:**
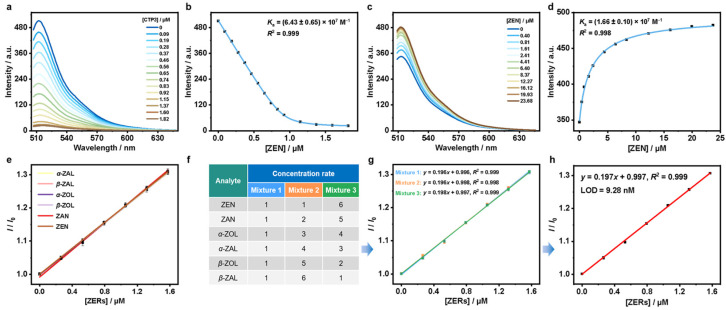
Direct fluorescence titration of Fl with CTP3, competing titration of CTP3•Fl reporter pair with ZEN in HEPES solution (10 mM, pH 7.4) at 25 °C, and the established standard curves of the tested ZERs. (**a**) Fluorescence titration of Fl (0.60 μM) directly at *λ*_ex_ = 500 nm and *λ_em_* = 513 nm using CTP3 (up to 1.82 μM). (**b**) The titration curve (*λ_em_* = 513 nm) of Fl (0.60 μM) with CTP3 acquired by a 1:1 binding model. (**c**) Competitive titration of CTP3•Fl (0.30/0.60 μM) reporter pair with ZEN (up to 23.68 μM). (**d**) The titration curve (*λ_em_* = 513 nm) of CTP3•Fl (0.30/0.60 μM) reporter pair with ZEN (up to 23.68 μM) obtained via a 1:1 binding model. (**e**) Calibration lines constructed by relative fluorescence intensities (*I*/*I_0_*) in comparison with the respective concentrations of ZEN, *α*-ZOL, *β*-ZOL, *α*-ZAL, *β*-ZAL, and ZAN in HEPES buffer (10 mM, pH 7.4, at 25 °C). (**f**) The mixed ZER solution in different concentration proportions. (**g**) Calibration lines created by plotting relative fluorescence intensities (*I*/*I_0_*) in opposition to the concentrations of the mixed ZERs with various proportions. (**h**) The integrated calibration line created by plotting *I*/*I_0_* against the concentrations of the mixed ZERs.

Given that classic macrocycles cannot effectively capture ZEN, we turned our attention to the novel macrocycles of BAs (CTP3, CQP3, and CPP3) with more flexible skeletons, more suitably sized cavities, and richer functional groups, improving non-covalent bonding interactions with ZEN. CTP3, CQP3, and CPP3 have been proven to share high affinities with ZEN, respectively, among which CTP3 exhibited the highest binding affinity (*K_a_* = (1.66 ± 0.10) × 10^7^ M^−1^), attributed to the flexible backbone, suitable cavities, and positively charged quaternary ammonium group for the overall encapsulation of ZEN (Figure 3a–d, Figures S10 and S11).

Importantly, the response consistency of CTP3 to ZAN, *α*-ZOL, *α*-ZAL, *β*-ZOL, *β*-ZAL, and ZEN was the key challenge in proving the feasibility of the established optical sensing based on CTP3. Fortunately, the synchronous recognition behaviors between CTP3 and the tested ZERs were observed. As depicted in Figure 3a–d and Figures S12–S16, the negligible fluctuation was shown in *K*_a_ from (1.66 ± 0.10) × 10^7^ to (1.69 ± 0.12) × 10^7^ M^−1^. Furthermore, the concentration-dependent signal responses of ZERs (*R*^2^ = 0.999) remained consistent across a single analyte and mixed analytes in varied proportions, which were displayed in Figure 3e–h and Table S1, paving the way for the supramolecular sensing of ZERs by the CTP3•Fl (0.40 µM/0.80 µM) reporter pair. LODs of the detected ZERs were, respectively, obtained below 10.77 nM by using the 3*σ*/slope method (Table S1). Each stock solution was prepared with a concentration of 100 μM, and the mixtures were prepared according to the three different molar ratios: [ZEN:ZAN:*α*-ZOL:*α*-ZAL:*β*-ZOL:*β*-ZAL] = 1:2:3:4:5:6, 1:1:1:1:1:1, and 6:5:4:3:2:1. The satisfactory linearity between *I*/*I_0_* (1.00–1.31) and ZERs level (0–1.57 µM) was acquired, y = 0.197x + 0.997 (*R*^2^ = 0.999), with a LOD of 9.28 nM (Figure 3h). In summary, the established method demonstrates high sensitivity and robustness for ZER sensing.

Compared with previously reported methods for the detection of ZEN, this strategy showed a lower LOD (9.28 nM), which indicated that the strategy had a better signal amplification effect and sensitivity [45,46]. Moreover, our recognition elements are more accessible, and the experimental operations are of greater simplicity.

### 3.3. Theoretical Calculation for Studying the CTP3•ZERs Complexes

DFT calculations allow for a thorough and detailed examination of the electronic structure and bonding behavior of host–guest pairs at the molecular level, which serve as a robust quantum mechanical framework and display the complex synergistic interactions that realize the highly sensitive detection of analytes. These calculations are conducive to elucidating the nature of the interactions, such as hydrogen bonding, electrostatic interactions, and Van der Waals forces, which are pivotal for the design and optimization of the supramolecular sensing platform. For further study on the molecular recognition behaviors, geometric optimization of the CTP3•ZERs complexes was conducted without symmetry constraints by undertaking density functional theory at the B3LYP/6-31G (d)/SMD (water) level for calculating binding energy. Grounded in the optimized geometry, the binding energy (ΔG) was computed to assess the stability of the host–guest complex of CTP3 with various analytes, which was proven to be as low as about −0.133 in a synchronous behavior, displaying thermodynamical stability of CTP3•ZERs complexes (Table S2), which agrees with the fluorescence titration results.

**Figure 4:**
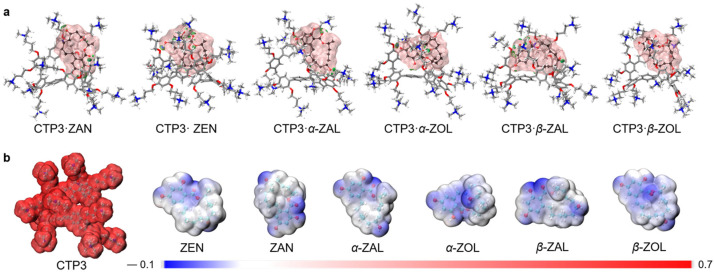
Elucidation of the CTP3•ZER complexes by theoretical calculation. (**a**) Optimized CTP3•ZER complexes at the B3LYP-D3(BJ)/6-31G(d)/SMD(water) level of theory. (**b**) MEP analysis of the molecular vdW surface of CTP3 and ZERs. The unit of MEP is hartree. δginter = 0.005 a.u. isosurfaces were colored by the sign of (*λ*_2_)ρ for the CTP3•ZER complexes.

The independent gradient model (IGMH) analysis primarily serves the purpose of elucidating the weak interactions existing among molecules [35]. An analysis additionally showed intermolecular noncovalent interactions between the different ZERs and CTP3, including the C-H···O, O-H···O, C-H···*π*, O-H···*π*, and Ar-H···O stacking and vdW interactions, showing similar molecular recognition behaviors (Figure 4a). In the IGMH analysis, –OH in ZEN formed a strong hydrogen bond (O–H···O) with the –C–O of in CTP3. In the IGMH analysis, the green isosurfaces further demonstrated multiple weak interactions between CTP3 and ZEN, including the C–H···*π* (–CH3 in CTP3 and *π* of benzene in ZEN), Ar–H···O (Ar–H in CTP4 and –C=O in ZEN), C–H···O (–C–O of in CTP3 and –C–H in ZEN), and vdw interactions (Figure 4a). Moreover, the MEP analysis of CTP3 and ZERs indicated that the electron-deficient regions (quaternary ammonium group) of CTP3 (depicted in red) and the electron-rich regions (carbonyl and hydroxyl) of ZERs (depicted in blue) come close to each other in a complementary electrostatic manner. (Figure 4b). Accordingly, multi-interaction synergies create robust binding complexes of CTP3•ZERs.

### 3.4. Study on the Inspection of ZER-Contaminated Cereals with CTP3•Fl Complex

In order to survey ZER pollution in different cereals, 30 batches of cereals (corn, Job’s tears, and malt) were pretreated with the established method and detected using the classic HPLC–FLD, which displayed the greatly fluctuating level of total ZERs, ranging from not detected (N.D.) to 4.290 ± 0.17 ppm. Additionally, none of the samples showed the presence of *α*-ZOL, *β*-ZOL, *α*-ZAL, and ZAN. Especially, Job’s tears were discovered to be contaminated with ZERs up to 0.7778 ± 0.01 ppm for ZEN and 4.290 ± 0.17 ppm for *β*-ZAL, both of which far exceeded the limits of GB 2761-2017 (ZEN level < 60 ppb) at the highest risk. Malt was verified to be at the lowest risk except for one batch (0.7644 ± 0.01 ppm for ZEN and N.D. for *β*-ZAL), which exceeded the limit of GB 2761-2017 [12]. For corn, one-half of the tested samples were found to be at high risk, whose ZEN contents ranged from 0.1450 ± 0.00 to 1.374 ± 0.02 ppm.

**Figure 5:**
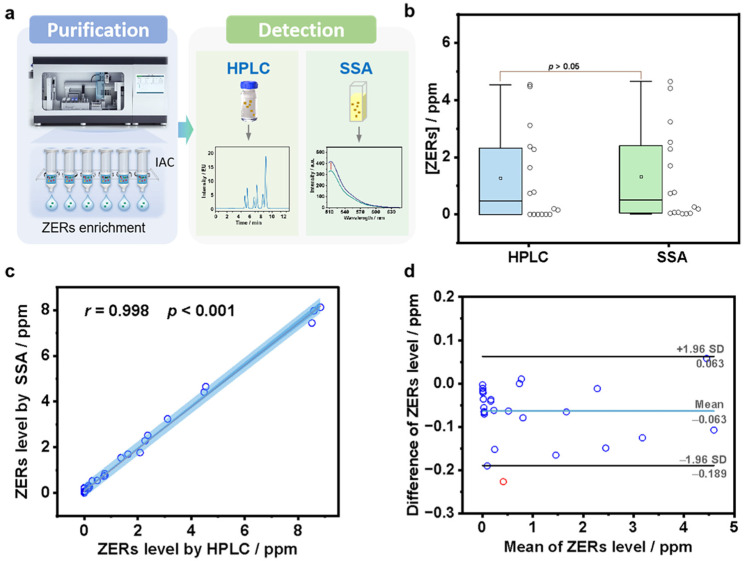
Application of ZER supramolecular sensing in the tested samples. (**a**) Purification procedure and detection methods of ZERs in samples. (**b**) Statistical analysis of ZER levels in the tested samples obtained by HPLC and SSA using Mann–Whitney U tests. (**c**) An excellent correlation (r = 0.998, *p* < 0.001) of ZER levels in the tested samples determined by HPLC and SSA. (**d**) Bland–Altman analysis of ZER levels in the tested samples between HPLC and SAA.

To evaluate the practicality and applicability of the supramolecular sensing assay (SSA), the established method was employed to detect the mentioned 30 batches of cereals (Figure 5a). The consistency of HPLC and sensing results were validated by Mann–Whitney U tests, Spearman’s correlation coefficients, and Bland–Altman analyses, respectively. ZER levels in 30 batches of samples determined by the different methods were not significantly different (Mann–Whitney U tests, *p* > 0.05) (Figure 5b, Table S3). As shown in Figure 5c, excellent correlations (Spearman’s correlation coefficients, r > 0.998, *p* < 0.001) were observed among the HPLC and SSA results. Furthermore, the Bland–Altman analysis showed satisfactory agreement except for one deviating point, exceeding 95% LoAs at a rate of 3.3% (1/30) (Figure 5d). The empirical results confirmed the feasibility of ZER sensing with the CTP3•Fl reporter pair.

Additionally, 10 supplementary batches of Job’s tears (JT11–JT20) were acquired through random sampling, and their ZER concentrations were measured using SSA. As illustrated in Table S4, six batches of samples (JT11–JT15, JT19) were quickly discriminated as danger samples, ranging from 0.07893 ± 0.01 to 8.126 ± 0.08 ppm in the content of ZERs. The remaining tested samples were classified as safe samples. These results were perfectly verified by HPLC–FLD, which confirmed the feasibility and applicability of the SSA of ZERs in cereals, realizing the high-throughput and facile inspection of ZER-contaminated cereals using optical sensing with the CTP3•Fl reporter pair.

## 4. Conclusions

In summary, simple and practical supramolecular sensing was accomplished to realize the monitoring of ZERs in cereals by the CTP3•Fl reporter pair, enabling the efficient identification of high-risk cereal samples. This approach based on extended biphen[n]arene macrocycles exhibits versatile characteristics, such as wide linear range, low LOD, and satisfactory stability, showing acceptable sample detection capabilities and application potential in the field of supramolecular sensing. This work not only reported an alternative approach for monitoring mycotoxin contamination in agricultural products but also broadened the application prospects of BA-based systems in food safety analysis, particularly for detecting low-abundance toxic substances through supramolecular signal transduction mechanisms.

## Figures and Tables

**Table 1:** The association constants of the complexes formed by macrocycle and fluorescent dye/ZEN.

Reporter Pair		*λ*_ex_ (*λ*_em_)/nm ^a^	*K*_a_/M^−1^ (Host•Dye)	*K*_a_/M^−1^ (Host•ZEN) ^b^	References
Macrocycle	Dye				
*β*-CD	2,6-TNS	350 (483)	(1.88 ± 0.36) × 10^3^	–	[33]
*γ*-CD	HPTS	405 (435)	(5.61 ± 0.28) × 10^1^	–	[33]
CB[6]	DSMI	450 (582)	(1.68 ± 0.26) × 10^5^	–	[33]
CB[7]	AO	450 (510)	(9.04 ± 0.54) × 10^4^	–	[33]
SC4A	LCG	368 (505)	(1.26 ± 0.13) × 10^7^	–	[33]
SC5A	LCG	368 (505)	(1.48 ± 0.21) × 10^6^	–	[33]
SC6A	LCG	368 (505)	(4.96 ± 0.62) × 10^7^	–	[33]
SAC4A	RhB	554 (576)	(9.26 ± 0.63) × 10^6^	–	[17]
SAC6A	RhB	554 (576)	(1.66 ± 0.46) × 10^6^	–	Our work
SAC8A	RhB	554 (576)	(7.68 ± 1.79) × 10^6^	–	Our work
GC5A	Fl	500 (513)	(5.0 ± 1.0) × 10^6^	–	[17]
CTP3	Fl	500 (513)	(6.43 ± 0.65) × 10^7^	(1.66 ± 0.10) × 10^7^	Our work
CQP3	Fl	500 (513)	(1.91 ± 0.16) × 10^7^	(3.65 ± 0.35) × 10^6^	Our work
CPP3	Fl	500 (513)	(1.11 ± 0.20) × 10^7^	(1.41 ± 0.15) × 10^6^	Our work

^a^ *λ*_ex_ represents the wavelength of fluorescence excitation. *λ*_em_ represents the maximum wavelength of fluorescence emission. ^b^ “−” represents no obvious binding affinity.

## Data Availability

The data in this study are available upon request from the corresponding author, yet remain privately restricted due to privacy and not publicly accessible.

## Supplementary Materials

The following supporting information can be downloaded at: https://www.mdpi.com/article/10.3390/foods14050863/s1, Figure S1. Competitive titration of *β*-CD•MB reporter pair with ZEN in 10 mM HEPES buffer solution (pH 7.4) at 25 °C; Figure S2. Competitive titration of ***γ***-CD•MB reporter pair with ZEN in 10 mM HEPES buffer solution (pH 7.4) at 25 °C; Figure S3. Competitive titration of CB[6]•DSMI reporter pair with ZEN 10 mM HEPES buffer solution (pH 7.4) at 25 °C; Figure S4. Competitive titration of CB[7]•AO reporter pair with ZEN in 10 mM HEPES buffer solution (pH 7.4) at 25 °C; Figure S5. Competitive titration of SC4A•LCG, SC5A•LCG, and SC6A•LCG reporter pairs with ZEN in 10 mM HEPES buffer solution (pH 7.4) at 25 °C; Figure S6. Competitive titration of SAC4A•RhB reporter pair with ZEN in 10 mM HEPES buffer solution (pH 7.4) at 25 °C; Figure S7. Direct fluorescence titration of RhB with SAC6A and competitive titration of SAC6A•RhB reporter pair with ZEN in 10 mM HEPES buffer solution (pH 7.4, 25 °C) at *λ*_ex_ = 554 nm and *λ*_em_ = 576 nm; Figure S8. Direct fluorescence titration of RhB with SAC8A and competitive titration of SAC8A•RhB reporter pair with ZEN in 10 mM HEPES buffer solution (pH 7.4, 25 °C) at *λ*_ex_ = 554 nm and *λ*_em_ = 576 nm; Figure S9. Competitive titration of GC5A•Fl reporter pair with ZEN in 10 mM HEPES buffer solution (pH 7.4) at 25 °C; Figure S10. Direct fluorescence titration of Fl with CQP3 and competitive titration of CQP3•Fl complex with ZEN in 10 mM HEPES buffer solution (pH 7.4, 25 °C), with *λ*_ex_ = 500 nm and *λ_em_* = 513 nm; Figure S11. Direct fluorescence titration of Fl with CPP3 and competitive titration of CPP3•Fl complex with ZEN in 10 mM HEPES buffer solution (pH 7.4, 25 °C), with *λ*_ex_ = 500 nm and *λ_em_* = 513 nm; Figure S12. Direct fluorescence titration of Fl with CTP3 and competitive titration of CTP3•Fl complex with *α*-ZOL in 10 mM HEPES buffer solution (pH 7.4, 25 °C), with *λ*_ex_ = 500 nm and *λ_em_* = 513 nm; Figure S13. Direct fluorescence titration of Fl with CTP3 and competitive titration of CTP3•Fl complex with *β*-ZOL in 10 mM HEPES buffer solution (pH 7.4, 25 °C), with *λ*_ex_ = 500 nm and *λ_em_* = 513 nm; Figure S14. Direct fluorescence titration of Fl with CTP3 and competitive titration of CTP3•Fl complex with *α*-ZALin 10 mM HEPES buffer solution (pH 7.4, 25 °C), with *λ*_ex_ = 500 nm and *λ_em_* = 513 nm; Figure S15. Direct fluorescence titration of Fl with CTP3 and competitive titration of CTP3•Fl reporter complex *β*-ZAL in 10 mM HEPES buffer solution (pH 7.4, 25 °C), with *λ*_ex_ = 500 nm and *λ_em_* = 513 nm; Figure S16. Direct fluorescence titration of Fl with CTP3 and competitive titration of CTP3•Fl complex with ZAN in 10 mM HEPES buffer solution (pH 7.4, 25 °C), with *λ*_ex_ = 500 nm and *λ_em_* = 513 nm; Table S1. The calibration curves of the different ZERs in 10 mM HEPES buffer solution (pH 7.4); Table S2. Calculated Gibbs free energy and binding energy of the complex of CTP3•ZERs; Table S3. The determination results of ZERs in 30 batches of cereals by HPLC and SSA; Table S4. The determination results of ZERs in 10 batches of cereals by SSA and HPLC.

## Abbreviations

The following abbreviations are used in this manuscript:

AO	Acridine Orange
BAs	Biphen[*n*]arenes
CB[6]	Cucurbit[6]uril
CB[7]	Cucurbit[7]uril
CB[8]	Cucurbit[8]uril
ChP	Chinese Pharmacopoeia
CI	Confidence Interval
CPP3	Quaternary Ammonium-functionalized Pentaphen[3]arene
CQP3	Quaternary Ammonium-functionalized Quaterphen[3]arene
CTP3	Quaternary Ammonium-functionalized Terphen[3]arene
DSMI	Trans-4-[4-(Dimethylamino)styryl]-1-methylpyridinium iodide
GC5A	Guanidinocalix[5]arene
HEPES	(4-(2-Hydroxyethyl)-1-piperazineethanesulfonic acid
HPLC–FLD	High-performance liquid chromatography equipped with a fluorescence detection system
HPTS	8-Hydroxypyren-1,3,6-trisulfonate
IARC	International Agency for Research on Cancer
IDA	Indicator Displacement Assay
IGMH	Independent Gradient Model based on Hirshfeld Partition
*K*_a_	Association Constant
LC–MS/MS	Liquid Chromatography–Tandem Mass Spectrometry
LoAs	Limits of Agreement
MB	Methylene Blue
MEP	Molecular Electrostatic Potential
N.D.	Not Detected
NR	Neutral Red
RhB	Rhodamine B
S.D.	Standard Deviation
SMD	Solvation Model
SSA	Supramolecular Sensing Assay
SAC6A	Sulfonated Azocalix[6]arene
SAC8A	Sulfonated Azocalix[8]arene
VMD	Visual Molecular Dynamics
ZAN	Zearalanone
ZEN	Zearalenone
ZERs	Zeranols
*α*-ZAL	*α*-Zearalanol
*α*-ZOL	*α*-Zearalenol
*β*-CD	*β*-Cyclodextrin
*β*-ZAL	*β*-Zearalanol
*β*-ZOL	*β*-Zearalenol
*γ*-CD	*γ*-Cyclodextrin
SC4A	*p*-Sulfonatocalix[4]arene
SC5A	*p*-Sulfonatocalix[5]arene
SC6A	*p*-Sulfonatocalix[6]arene
