# Supplementary material for: Rapid Detection of Zeranol Contamination in Cereals Using a Quaternary Ammonium-Functionalized Terphen[3]arene-Based Optical Sensor
Source: Foods. 2025 Mar 3;14(5):863. doi: 10.3390/foods14050863 (PMC11898769; doi:10.3390/foods14050863)
Supplement: Supplementary file 1 [file foods-14-00863-s001.zip › foods-3433646-supplementary.pdf]

Rapid Detection of Zeranol Contamination in Cereals Using a Quaternary Ammonium-Functionalized Terphen[3]arene-Based Optical Sensor

Danni Wang [1,†], Mengyu Ye [1,†], Huijuan Yu [1,2], Kejing Niu [1], Chunju Li [3], Dong-Sheng Guo [4,*] and Yuefei Wang [1,2,*]

[1] State Key Laboratory of Chinese Medicine Modernization, Tianjin University of Traditional Chinese Medicine, Tianjin 301617, China
[2] Haihe Laboratory of Modern Chinese Medicine, Tianjin 301617, China
[3] Key Laboratory of Inorganic-Organic Hybrid Functional Material Chemistry, Ministry of Education, Tianjin Key Laboratory of Structure and Performance for Functional Molecules, College of Chemistry, Tianjin Normal University, Tianjin 300387, China
[4] State Key Laboratory of Elemento-Organic Chemistry, Key Laboratory of Functional Polymer Materials (Ministry of Education), Frontiers Science Center for New Organic Matter, Collaborative Innovation Center of Chemical Science and Engineering, College of Chemistry, Nankai University, Tianjin 300071, China
* Correspondence: dshguo@nankai.edu.cn (D.-S.G.); wangyf0622@tjutcm.edu.cn (Y.W.)
† These authors contributed equally to this work.

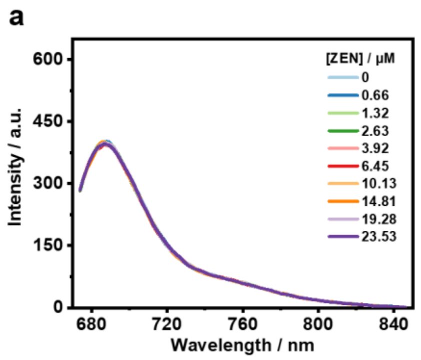

Figure S1. Competitive titration of β-CD•MB reporter pair with ZEN in 10 mM HEPES buffer solution (pH 7.4) at 25 °C. (a) Fluorescence spectra of MB (10.00 μM) titrated by ZEN (up to 23.53 μM). (b) Competitive titration of β-CD•MB (1.00 mM / 10.00 μM) reporter pair with ZEN (up to 23.53 μM).

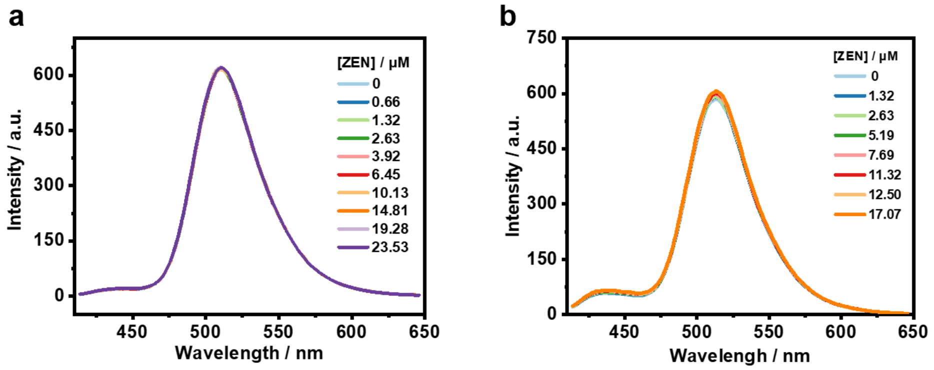

Figure S2. Competitive titration of γ-CD•HPTS reporter pair with ZEN in 10 mM HEPES buffer solution (pH 7.4) at 25 °C. (a) Fluorescence spectra of HPTS (10.00 μM) titrated by ZEN (up to 23.53 μM). (b) Competitive titration of γ-CD•HPTS (10.00 mM / 10.00 μM) reporter pair with AFB1 transformant (up to 17.07 μM).

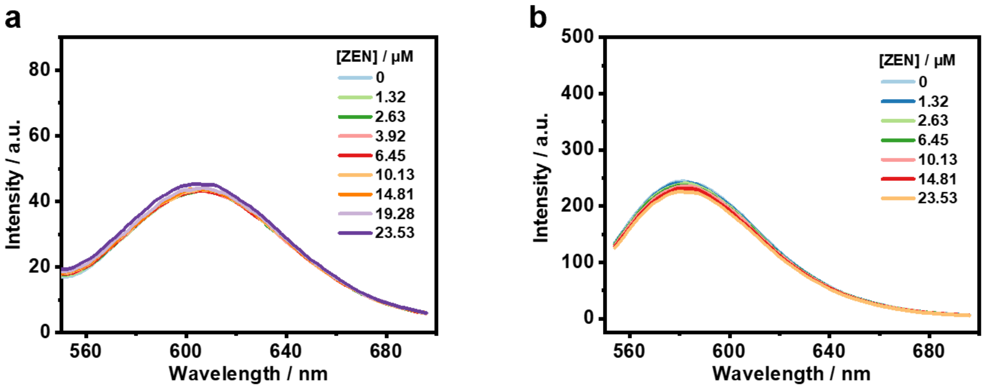

Figure S3. Competitive titration of CB[6]•DSMI reporter pair with ZEN 10 mM HEPES buffer solution (pH 7.4) at 25 °C. (a) Fluorescence spectra of DSMI (1.00 μM) titrated by ZEN (up to 23.53 μM). (b) Competitive titration of CB[6]•DSMI (8.00 μM / 1.00 μM) reporter pair with ZEN (up to 23.53 μM).

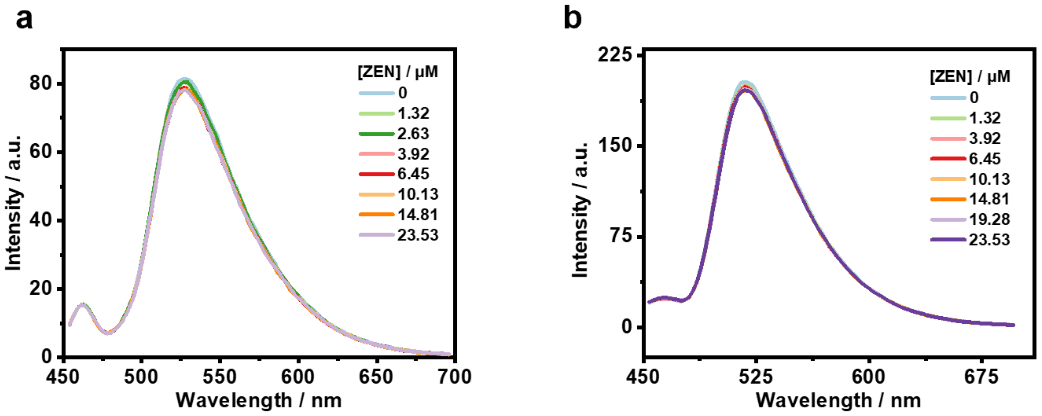

Figure S4. Competitive titration of CB[7]•AO reporter pair with ZEN in 10 mM HEPES buffer solution (pH 7.4) at 25 °C. (a) Fluorescence spectra of AO (0.50 μM) titrated by ZEN (up to 23.53 μM). (b) Competitive titration of CB[7]•AO (15.00 μM / 0.50 μM) reporter pair with ZEN (up to 23.53 μM).

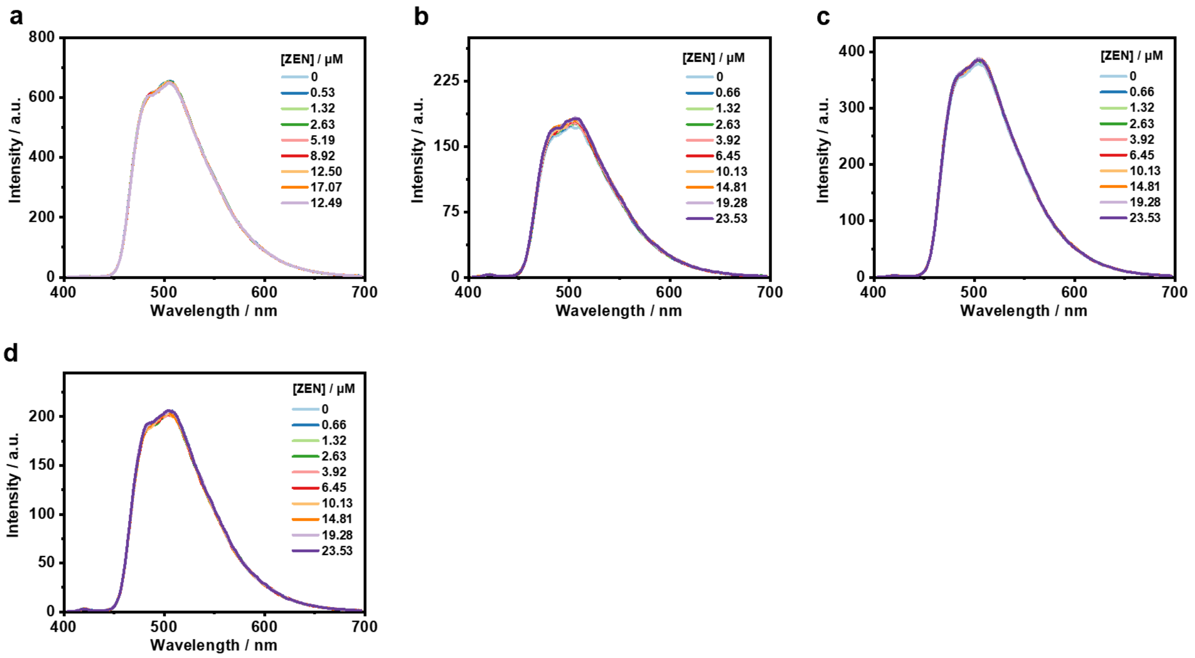

Figure S5. Competitive titration of SC4A•LCG, SC5A•LCG, and SC6A•LCG reporter pairs with ZEN in 10 mM HEPES buffer solution (pH 7.4) at 25 °C. (a) Fluorescence spectra of LCG (0.50 μM) titrated by ZEN (up to 12.49 μM). (b) Competitive titration of SC4A•LCG (0.50 μM / 0.50 μM) reporter pair with ZEN (up to 23.53 μM). (c) Competitive titration of SC5A•LCG (1.00 μM / 1.00 μM) reporter pair with ZEN (up to 23.53 μM). (d) Competitive titration of SC6A•LCG (1.00 μM / 1.00 μM) reporter pair with ZEN (up to 23.53 μM).

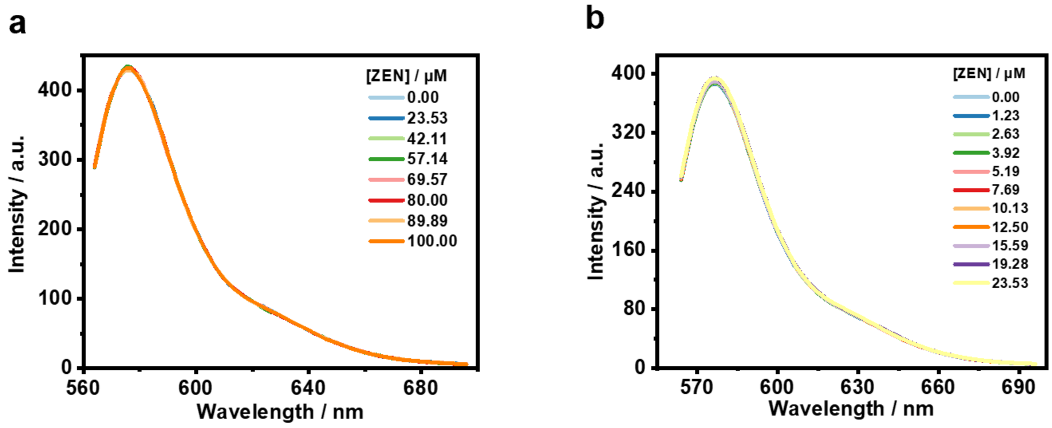

Figure S6. Competitive titration of SAC4A•RhB reporter pair with ZEN in 10 mM HEPES buffer solution (pH 7.4) at 25 °C. (a) Fluorescence spectra of RhB (0.80 μM) titrated by ZEN (up to 100.00 μM). (b) Competitive titration of SAC4A•RhB (1.00 μM / 0.80 μM) reporter pair with ZEN (up to 23.53 μM).

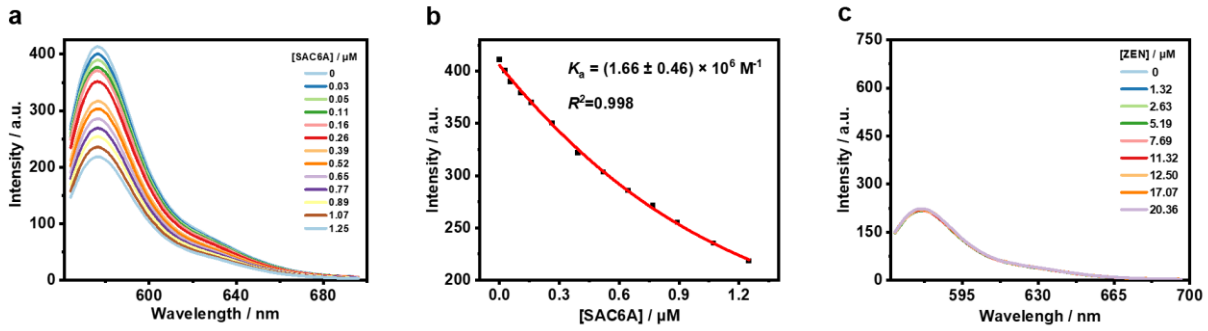

Figure S7. Direct fluorescence titration of RhB with SAC6A and competitive titration of SAC6A•RhB reporter pair with ZEN in 10 mM HEPES buffer solution (pH 7.4, 25 °C) at λ_{ex} = 554 nm and λ_{em} = 576 nm. (a) Direct fluorescence titration of RhB (0.80 µM) with SAC6A (up to 1.25 µM). (b) The titration curve (λ_{em} = 576 nm) of RhB (0.80 µM) with SAC6A acquired by a 1:1 binding model. (c) Competitive titration of SAC6A•RhB (20.00 µM / 0.80 µM) reporter pair with ZEN (up to 15.14 µM).

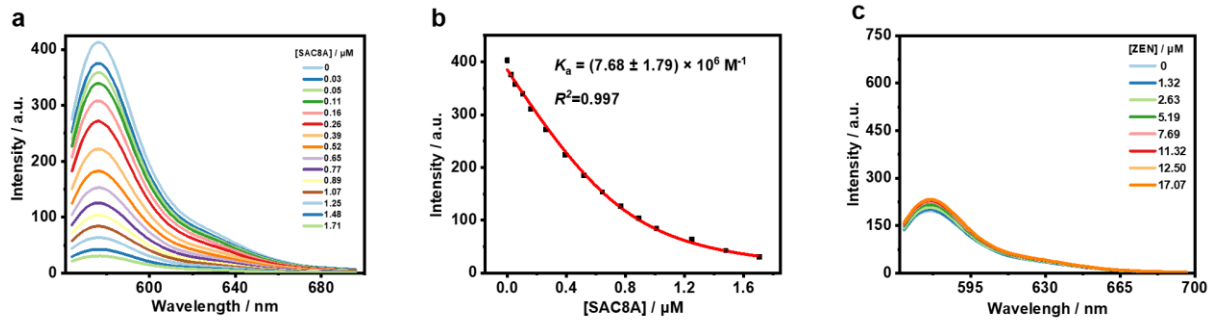

Figure S8. Direct fluorescence titration of RhB with SAC8A and competitive titration of SAC8A•RhB reporter pair with ZEN in 10 mM HEPES buffer solution (pH 7.4, 25 °C) at λ_{ex} = 554 nm and λ_{em} = 576 nm. (a) Direct fluorescence titration of RhB (0.80 µM) with SAC8A (up to 1.71 µM). (b) The titration curve (λ_{em} = 576 nm) of RhB (0.80 µM) with SAC8A acquired by a 1:1 binding model. (c) Competitive titration of SAC8A•RhB (20.00 µM / 0.80 µM) reporter pair with ZEN (up to 17.07 µM).

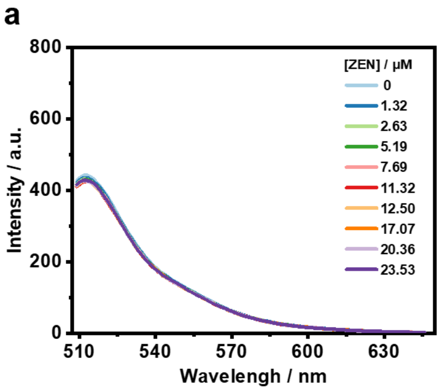

Figure S9. Competitive titration of GC5A•Fl reporter pair with ZEN in 10 mM HEPES buffer solution (pH 7.4) at 25 °C. (a) Competitive titration of GC5A•Fl (15.00 μM / 0.80 μM) reporter pair with ZEN (up to 23.53 μM).

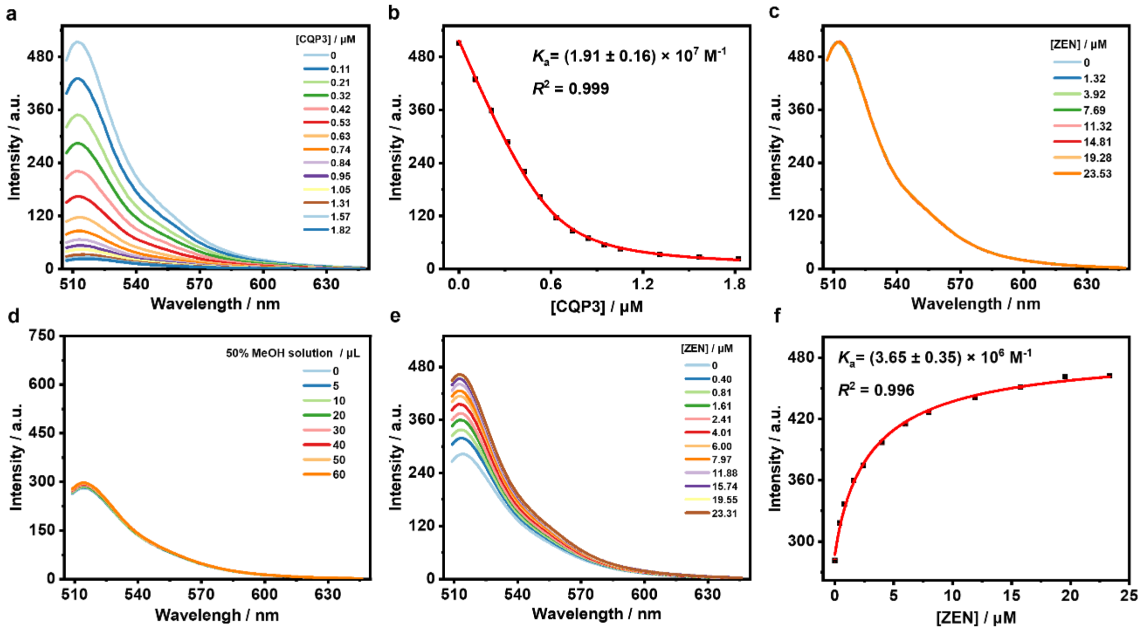

Figure S10. Direct fluorescence titration of Fl with CQP3 and competitive titration of CQP3•Fl complex with ZEN in 10 mM HEPES buffer solution (pH 7.4, 25 °C) at λ_{ex} = 500 nm and λ_{em} = 513 nm. (a) Direct fluorescence titration of Fl (0.60 μM) with CQP3 (up to 1.82 μM). (b) The titration curve (λ_{em} = 513 nm) of Fl (0.60 μM) with CQP3 acquired by a 1:1 binding model. (c) Fluorescence spectra of Fl (0.60 μM) titrated by ZEN (up to 23.53 μM). (d) Competitive titration of CQP3•Fl (0.30/0.60 μM) reporter pair titrated by 50% methanol solution (up to 60 μL). (e) Competitive titration of CQP3•Fl (0.30/0.60 μM) reporter pair with ZEN (up to 23.31 μM). (f) The titration curve (λ_{em} = 513 nm) of CQP3•Fl (0.30/0.60 μM) reporter pair with ZEN (up to 23.31 μM) acquired by a 1:1 binding model.

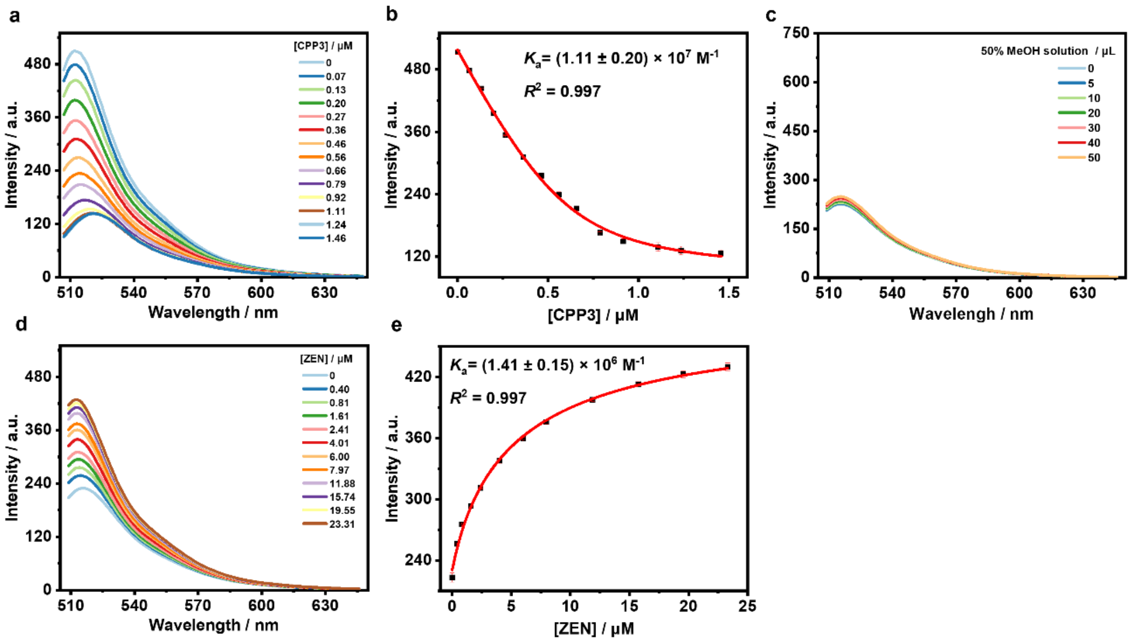

Figure S11. Direct fluorescence titration of Fl with CPP3 and competitive titration of CPP3•Fl complex with ZEN in 10 mM HEPES buffer solution (pH 7.4, 25 °C) at λ_{ex} = 500 nm and λ_{em} = 513 nm. (a) Direct fluorescence titration of Fl (0.60 μM) with CPP3 (up to 1.46 μM). (b) The titration curve (λ_{em} = 513 nm) of Fl (0.60 μM) with CPP3 acquired by a 1:1 binding model. (c) Competitive titration of CPP3•Fl (0.50/0.60 μM) reporter pair titrated by 50% methanol solution (up to 50 μL). (d) Competitive titration of CPP3•Fl (0.50/0.60 μM) reporter pair with ZEN (up to 23.31 μM). (e) The titration curve (λ_{em} = 513 nm) of CPP3•Fl (0.50/0.60 μM) reporter pair with ZEN (up to 23.31 μM) acquired by a 1:1 binding model.

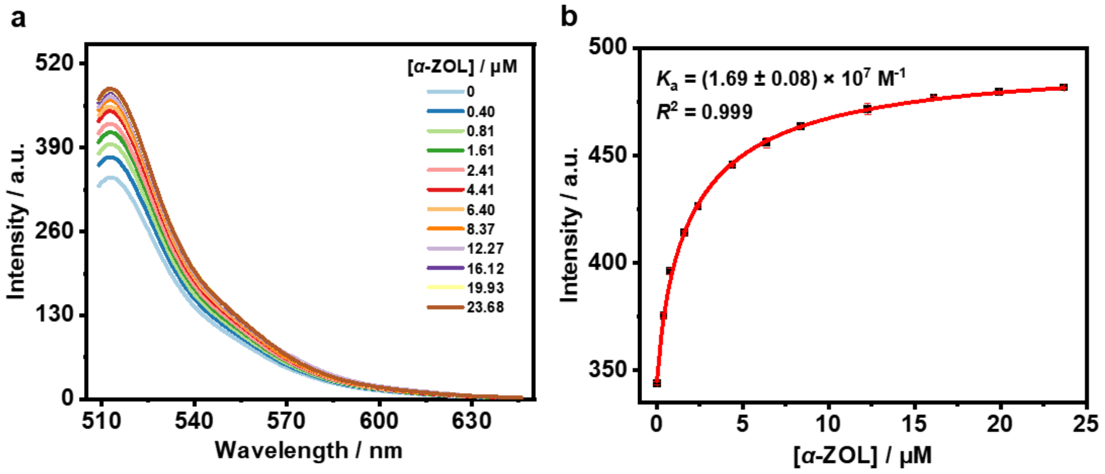

Figure S12 Direct fluorescence titration of Fl with CTP3 and competitive titration of CTP3•Fl complex with *α*-ZOL in HEPES buffer solution (10 mM, pH 7.4, 25 °C) at λ_{ex} = 554 nm and λ_{em} = 576 nm. (a) Competitive titration of CTP3•Fl (0.30/0.60 μM) reporter pair with *α*-ZOL (up to 23.68 μM). (b) The titration curve (λ_{em} = 513 nm) of CTP3•Fl (0.30/0.60 μM) reporter pair with *α*-ZOL (up to 23.68 μM) acquired by a 1:1 binding model.

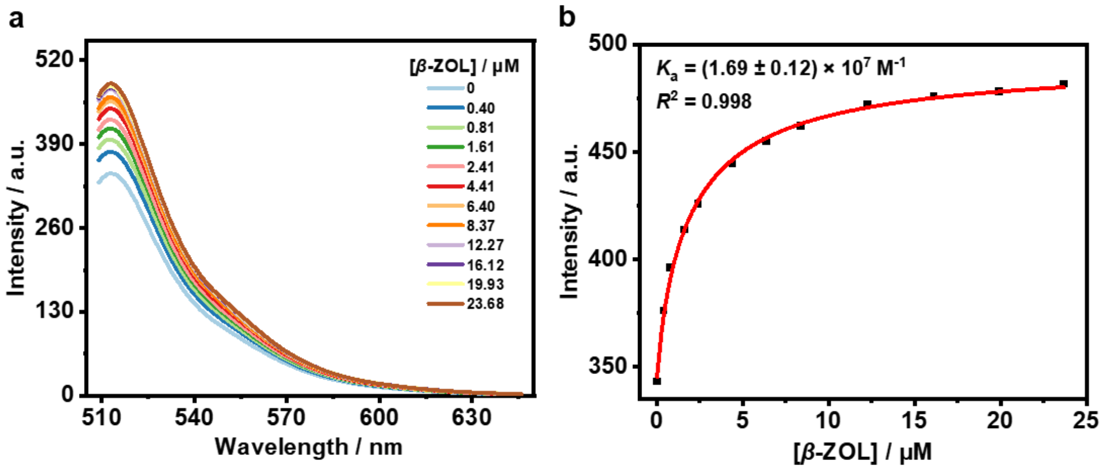

Figure S13 Direct fluorescence titration of Fl with CTP3 and competitive titration of CTP3•Fl complex with *β*-ZOL in 10 mM HEPES buffer solution (pH 7.4, 25 °C) at λ_{ex} = 554 nm and λ_{em} = 576 nm. (a) Competitive titration of CTP3•Fl (0.30/0.60 μM) reporter pair with *β*-ZOL (up to 23.68 μM). (b) The titration curve (λ_{em} = 513 nm) of CTP3•Fl (0.30/0.60 μM) reporter pair with *β*-ZOL (up to 23.68 μM) acquired by a 1:1 binding model.

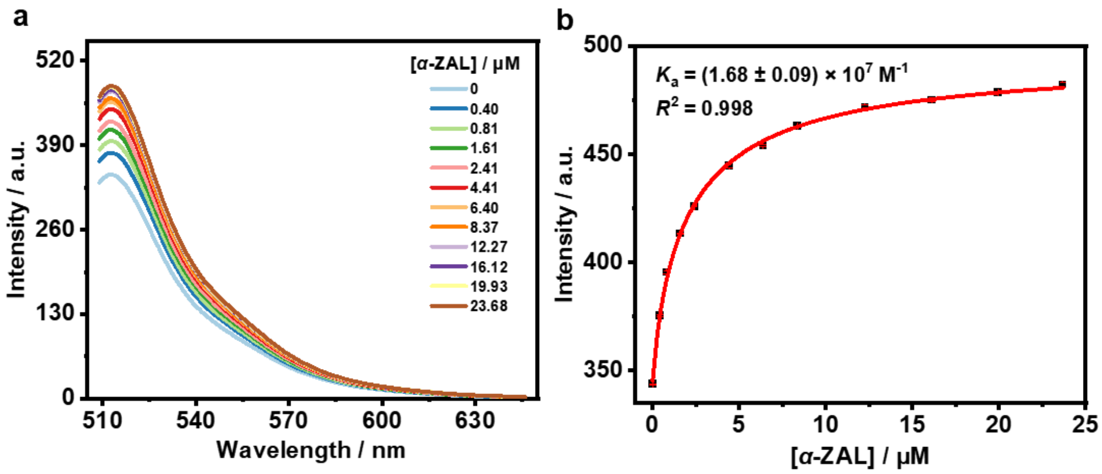

Figure S14 Direct fluorescence titration of Fl with CTP3 and competitive titration of CTP3•Fl complex with *α*-ZAL in 10 mM HEPES buffer solution (pH 7.4, 25 °C) at λ_{ex} = 554 nm and λ_{em} = 576 nm. (a) Competitive titration of CTP3•Fl (0.30/0.60 μM) reporter pair with *α*-ZAL (up to 23.68 μM). (b) The titration curve (λ_{em} = 513 nm) of CTP3•Fl (0.30/0.60 μM) reporter pair with *α*-ZAL (up to 23.68 μM) acquired by a 1:1 binding model.

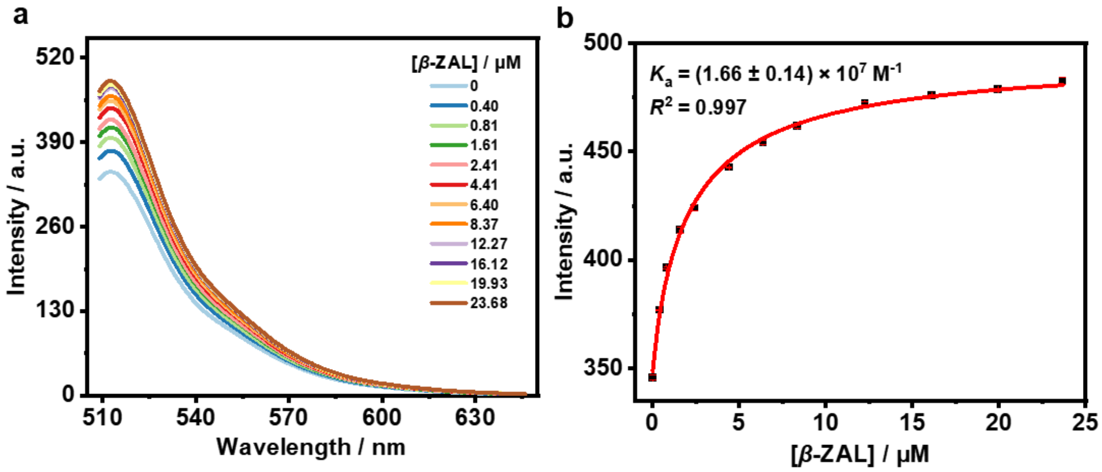

Figure S15 Direct fluorescence titration of Fl with CTP3 and competitive titration of CTP3•Fl complex with *β*-ZAL in 10 mM HEPES buffer solution (pH 7.4, 25 °C) at λ_{ex} = 554 nm and λ_{em} = 576 nm. (a) Competitive titration of CTP3•Fl (0.30/0.60 μM) reporter pair with *β*-ZAL (up to 23.68 μM). (b) The titration curve (λ_{em} = 513 nm) of CTP3•Fl (0.30/0.60 μM) reporter pair with *β*-ZAL (up to 23.68 μM) acquired by a 1:1 binding model.

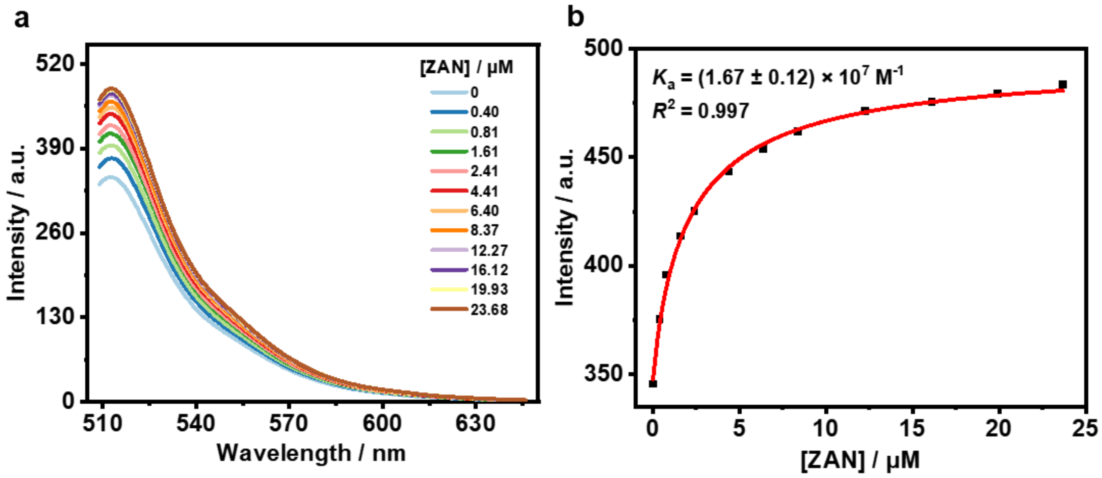

Figure S16 Direct fluorescence titration of Fl with CTP3 and competitive titration of CTP3•Fl complex with ZAN in 10 mM HEPES buffer solution (pH 7.4, 25 °C) at λ_{ex} = 554 nm and λ_{em} = 576 nm. (a) Competitive titration of CTP3•Fl (0.30/0.60 μM) reporter pair with ZAN (up to 23.68 μM). (b) The titration curve (λ_{em} = 513 nm) of CTP3•Fl (0.30/0.60 μM) reporter pair with ZAN (up to 23.68 μM) acquired by a 1:1 binding model.

Table S1 The calibration curves of the different ZERs in HEPES buffer (10 mM, pH 7.4)

Analytes	Regression equation	r^2	Linear range (μM)	LOD (nM)
α-ZOL	$y = 0.196\ x + 0.996$	0.999	0 ~ 1.57	6.52
β-ZOL	$y = 0.195\ x + 0.998$	0.999	0 ~ 1.57	5.10
α-ZAL	$y = 0.198\ x + 0.996$	0.999	0 ~ 1.57	10.07
β-ZAL	$y = 0.197\ x + 0.996$	0.999	0 ~ 1.57	9.09
ZAN	$y = 0.198\ x + 0.996$	0.999	0 ~ 1.57	10.77

Table S2. Calculated Gibbs free energy and binding energy of the complex of CTP3•ZERs. The energy units are hartree.

Complex	G_{H}	G_{G}	G_{HG}	ΔG
CTP3•ZAN	−7676.692	−6600.031	−1076.533	−0.128
CTP3•ZEN	−7675.458	−6600.016	−1075.309	−0.133
CTP3•*α*-ZAL	−7677.881	−6600.030	−1077.731	−0.120
CTP3•*α*-ZOL	−7676.645	−6600.032	−1076.499	−0.114
CTP3•*β*-ZAL	−7677.899	−6600.021	−1077.724	−0.154
CTP3•*β*-ZOL	−7676.650	−6600.015	−1076.500	−0.135

Table S3 The determination results of ZERs in 30 batches cereals by HPLC and SSA

Sample Number	ZERs content（ppm）	
	HPLC	SSA
C1	N.D.[a]	0.06473 ± 0.01
C2	0.2983 ± 0.00	0.5250 ± 0.01
C3	N.D.	N.D.
C4	1.374 ± 0.02	1.539 ± 0.13
C5	N.D.	0.03577 ± 0.07
C6	N.D.	0.06752 ± 0.11
C7	0.1647 ± 0.00	0.3167 ± 0.08
C8	0.1450 ± 0.00	0.1854 ± 0.03

C9	N.D.	N.D.
C10	0.4855 ± 0.03	0.5489 ± 0.09
M1	N.D.	N.D.
M2	0.7644 ± 0.01	0.8436 ± 0.01
M3	N.D.	N.D.
M4	N.D.	0.1901 ± 0.12
M5	N.D.	0.03577 ± 0.07
M6	N.D.	0.06752 ± 0.11
M7	N.D.	0.07040 ± 0.00
M8	N.D.	N.D.
M9	N.D.	N.D.
M10	N.D.	0.03618 ± 0.01
JT1	4.711 ± 0.11	4.649 ± 0.02
JT2	4.575 ± 0.18	4.412 ± 0.40
JT3	2.488 ± 0.10	2.522 ± 0.20
JT4	0.1958 ± 0.00	0.2578 ± 0.11
JT5	1.633 ± 0.02	1.698 ± 0.13
JT6	3.114 ± 0.10	3.239 ± 0.12
JT7	2.274 ± 0.10	2.286 ± 0.09
JT8	0.7341 ± 0.00	0.7344 ± 0.08
JT9	0.1495 ± 0.00	0.1865 ± 0.03
JT10	0.7778 ± 0.01	0.7672 ± 0.12

[a] N.D. indicates not detected.

Table S4. The determination results of ZERs in 10 batches of cereals by SSA and HPLC.

Sample Number	ZERs content (ppm)	
	HPLC	SSA
JT11	8.509 ± 0.35	7.437 ± 0.04
JT12	2.085 ± 0.12	1.767 ± 0.04
JT13	8.829 ± 0.21	8.126 ± 0.08
JT14	8.582 ± 0.14	7.972 ± 0.03
JT15	0.1271 ± 0.00	0.1915 ± 0.27
JT16	N.D. [a]	N.D.
JT17	N.D.	N.D.
JT18	N.D.	N.D.
JT19	0.03620 ± 0.06	0.07893 ± 0.01
JT20	N.D.	N.D.

[a] N.D. indicates not detected.
